# Comparison of Accelerometry-Based Measures of Physical Activity: Retrospective Observational Data Analysis Study

**DOI:** 10.2196/38077

**Published:** 2022-07-22

**Authors:** Marta Karas, John Muschelli, Andrew Leroux, Jacek K Urbanek, Amal A Wanigatunga, Jiawei Bai, Ciprian M Crainiceanu, Jennifer A Schrack

**Affiliations:** 1 Department of Biostatistics Bloomberg School of Public Health Johns Hopkins University Baltimore, MD United States; 2 Department of Biostatistics and Informatics Colorado School of Public Health University of Colorado Aurora, CO United States; 3 Center on Aging and Health, Division of Geriatric Medicine and Gerontology Department of Medicine, School of Medicine Johns Hopkins University Baltimore, MD United States; 4 Department of Epidemiology Bloomberg School of Public Health Johns Hopkins University Baltimore, MD United States

**Keywords:** accelerometry, actigraphy, activity counts, wearable computing, monitor-independent movement summary, MIMS, physical activity, aging, older adult population, wearable device, health monitoring, digital health, wearable technology, health technology

## Abstract

**Background:**

Given the evolution of processing and analysis methods for accelerometry data over the past decade, it is important to understand how newer summary measures of physical activity compare with established measures.

**Objective:**

We aimed to compare objective measures of physical activity to increase the generalizability and translation of findings of studies that use accelerometry-based data.

**Methods:**

High-resolution accelerometry data from the Baltimore Longitudinal Study on Aging were retrospectively analyzed. Data from 655 participants who used a wrist-worn ActiGraph GT9X device continuously for a week were summarized at the minute level as ActiGraph activity count, monitor-independent movement summary, Euclidean norm minus one, mean amplitude deviation, and activity intensity. We calculated these measures using open-source packages in R. Pearson correlations between activity count and each measure were quantified both marginally and conditionally on age, sex, and BMI. Each measures pair was harmonized using nonparametric regression of minute-level data.

**Results:**

Data were from a sample (N=655; male: n=298, 45.5%; female: n=357, 54.5%) with a mean age of 69.8 years (SD 14.2) and mean BMI of 27.3 kg/m2 (SD 5.0). The mean marginal participant-specific correlations between activity count and monitor-independent movement summary, Euclidean norm minus one, mean amplitude deviation, and activity were r=0.988 (SE 0.0002324), r=0.867 (SE 0.001841), r=0.913 (SE 0.00132), and r=0.970 (SE 0.0006868), respectively. After harmonization, mean absolute percentage errors of predicting total activity count from monitor-independent movement summary, Euclidean norm minus one, mean amplitude deviation, and activity intensity were 2.5, 14.3, 11.3, and 6.3, respectively. The accuracies for predicting sedentary minutes for an activity count cut-off of 1853 using monitor-independent movement summary, Euclidean norm minus one, mean amplitude deviation, and activity intensity were 0.981, 0.928, 0.904, and 0.960, respectively. An R software package called SummarizedActigraphy, with a unified interface for computation of the measures from raw accelerometry data, was developed and published.

**Conclusions:**

The findings from this comparison of accelerometry-based measures of physical activity can be used by researchers and facilitate the extension of knowledge from existing literature by demonstrating the high correlation between activity count and monitor-independent movement summary (and other measures) and by providing harmonization mapping.

## Introduction

The use of accelerometry-based activity monitors has become increasingly popular in research studies because they provide noninvasive objective measures of physical activity, and with these monitors, physical activity data can be collected continuously for extended periods of time [1]. Modern wearable accelerometers measure acceleration of a body at a high frequency (typically 10-100 Hz). These raw data are then typically aggregated into fixed-time epochs. Yet, the choice of epoch-based measures varies across studies. For example, the Baltimore Longitudinal Study on Aging [2] used wrist-worn accelerometers and summarized data using activity counts, a measure proposed and implemented by ActiGraph [3]. Monitor-independent movement summary [4] was used for wrist-worn accelerometry data collected for the National Health and Nutrition Examination Survey (NHANES) 2011-2014 [5]. The UK Biobank study [6] used wrist-worn accelerometers and Euclidean norm minus one [7]. Additional summary measures of acceleration are mean amplitude deviation [8] and activity intensity [9].

Given the evolution of processing and analysis methods for accelerometry data over the past decade, it is important to know how new summary measures compare with established measures. Harmonizing, or mapping, values of physical activity summaries derived from different algorithms enables knowledge from the thousands of manuscripts that have been published using ActiGraph activity count [10] (and for which no repository or access to raw accelerometry data is currently available).

In this study, we aimed to (1) provide simple summaries of associations between pairs of minute-level measures (ActiGraph activity count and monitor-independent movement summary, Euclidean norm minus one, mean amplitude deviation, activity intensity) and a guide for the strength of these associations in subgroups defined by demographic information; (2) provide a mapping between any 2 physical activity summary measures considered; (3) derive cut-points of open-source physical activity measures that correspond to established cut-points to estimate time spent in different physical activity intensities for activity count.

## Methods

### Study Design and Population

We conducted a retrospective data analysis study using data collected as part of the National Institute on Aging’s Baltimore Longitudinal Study of Aging (BLSA) from participants who were community-dwelling volunteers free of all major chronic conditions and cognitive and functional impairment at the time of enrollment [2]. The data used in this work were from participants who agreed to wear an accelerometer between July 2015 and January 2019 .

### Ethics Approval and Consent to Participate

The BLSA study protocol has ongoing approval from the Institutional Review Board (IRB) of the National Institute of Environmental Health Science, National Institutes of Health ("Early Markers of Alzheimer’s Disease [BLSA]", IRB No. 2009-074). Informed written consent was obtained from all participants.

### Accelerometry Data Collection and Export

Data had been collected with a triaxial accelerometer (ActiGraph GT9X Link; range: ±8 *g*; frequency: 80 Hz). Participants had been instructed to wear the accelerometer on their nondominant wrist for 7 days, except for periods of extended swimming or bathing. The ActiLife software (version 6.13.4) was used to (1) export data into GT3X file format, (2) derive and export minute-level ActiGraph activity count as CSV files, and (3) export raw acceleration data (in *g*) as three-dimensional time series with subsecond-level timestamps into CSV files. The ActiLife's low-frequency extension (a filtering option that decreases the lower end of the intensity threshold to increase sensitivity to low-intensity movements) was used based on recommendations and findings of greater comparability with older ActiGraph devices (model 7164) [11]. Hereon, *activity count* is used to denote ActiGraph activity count.

### Raw Accelerometry Data Quality Control

We used 3 raw data quality check flags (Multimedia Appendix 1) adapted from a set of 9 flags in the NHANES protocol [12]. The selected flags subset represents intuitive flags that are meant to “determine signal patterns that were unlikely to be a result of human movement” but are not aimed at identifying nonwear [12]. A raw data observation was valid if none of the 3 flags were triggered and invalid otherwise.

### Summary Measures of Raw Accelerometry Data

Commonly used minute-level measures—monitor-independent movement summary, Euclidean norm minus one, mean amplitude deviation, and activity intensity (Multimedia Appendix 2 [4, 7-9])—were calculated using raw accelerometry data. With R software (version 3.6.3; The R Project), we developed and used SummarizedActigraphy R package to compute the measures. SummarizedActigraphy is a package that provides a unified data interface to compute a range of measures; it references original software for computing monitor-independent movement summary (R package: MIMSunit [13], version 0.9.2) and calibrating data for computation of Euclidean norm minus one (R package: GGIR [14], version 2.3).

### Minute-Level Accelerometry Data Preprocessing

We defined minute-level data flags that represented whether the device was being worn or not using the *get_wear_flag* method (R package: arctools [15]; version 1.1.4), which implements a wear status detection algorithm based on activity count data [16]. A given minute was classified as nonwear if it belonged to a 90-minute interval with consecutive 0-values in activity count data; otherwise, the minute was classified as wear. A given minute was valid if no raw data-level quality control flags had been triggered within the minute and it had been classified as wear, and invalid otherwise. A valid day was defined as a day (12:00 AM to 11:59 PM) with no more than 10% (144 minutes) [17] invalid minutes. Only data from participants who had at least 3 valid days of data, and only data from valid days, were included in further preprocessing and analyses.

Activity count, monitor-independent movement summary, Euclidean norm minus one, mean amplitude deviation, and activity intensity data were winsorized [18] to reduce the effect of extreme values in the data set, by computing the measure-specific 0.999 quantile and then using it to replace values that exceeded this quantile.

A separate data set was constructed with imputed data, using a method described in [19]. Imputation was conducted separately for each measure: invalid minutes were replaced with corresponding values from smoothed time series produced using functional principal component analysis of the original participant- and day-specific minute-level time series (in which invalid minutes data had been denoted by *NA*). We used the *fpca.face* method (R package: refund [20], version 0.1.23) for functional principal component analysis due to its computational speed and given the large volume of data. The resulting data set was used in the summary of daily sums of measures values and in our application example where data without missing values were needed.

### Statistical Data Analysis

The mean daily sums of minute-level measures were computed for each participant and then aggregated (mean and SD; median and range) across participants.

Pearson correlation coefficients for 4 pairs of measures—activity count and monitor-independent movement summary, activity count and Euclidean norm minus one, activity count and mean amplitude deviation, and activity count and activity intensity—were computed for each participant. For each pair, mean correlations and standard errors were quantified using intercept-only linear regression with participant-specific correlation as the outcome. The effects of demographic characteristics (covariates: age, BMI, and sex) on correlations were estimated using adjusted linear regression with participant-specific correlation as the outcome and α=.05 to determine the statistical significance of coefficients. This procedure was repeated for secondary analyses with a subsample (participants’ age ≤65 years).

### Harmonization

#### Mapping

To derive the harmonization mapping, relationships were estimated using generalized additive modeling for each pair of measures. The generalized additive models were chosen to allow flexible adaptation to the data rather than imposing a particular functional form of the fit. In each model, the outcome was a minute-level measure (monitor-independent movement summary, or Euclidean norm minus one, or mean amplitude deviation, or activity intensity), and a smooth term of minute-level activity count was set as a predictor. For the smooth term, cubic regression splines with a basis dimension equal to 30 were used to allow a flexible relationship between the measure and activity count. Models were estimated with nonparametric smoothing (method: *gam*; R package: mgcv [21], version 1.8.34). Smoothness of the nonlinear effects was enforced via a second derivative penalty, and parameter selection was performed using cross-validation [22]. Data from all participants' valid minutes were used in the model fitting except for minutes, which had activity count values equal 0. The activity count=0 exclusion was motivated by a large proportion of zero values, and the need to estimate the relation for small activity count values without it being inflated by the large number of zeros. Relationships were estimated as strictly monotonic (without monotonicity having been constrained explicitly). The generalized additive model was used to provide values for 2-way mapping between activity count and each measure. All measurements were mapped into activity count, where 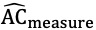
(*x*) represents the activity count value estimated by mapping the *x* value of a measure, where *measure* represents monitor-independent movement summary, Euclidean norm minus one, mean amplitude deviation, or activity intensity.

#### Evaluation

To assess mapping accuracy in estimating physical activity volume statistics, total activity count (the sum of minute-level activity count values from a day) was computed for each participant, using activity count data and 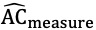, and the difference was defined the estimation error. Estimation error was summarized by calculating mean percentage error (MPE), mean absolute percentage error (MAPE), median percentage error, and median absolute percentage error for each participant and aggregated across participants (mean and SD).

To assess whether mapping accuracy depended on participant activity level, MPE values were plotted against the participant's average total activity count.

The utility of the mapping for classifying minutes into various activity intensity classes was assessed. We used activity count cut-offs derived to (1) separate sedentary and active minutes in data collected with a sensor worn on nondominant wrist in older adults [23], (2) separate sedentary from light and (3) light from moderate-to-vigorous activity intensity levels in data collected with a sensor worn on a nondominant wrist in young to older adults [24]. In the classification task, for each minute, the true value was defined based on whether activity count > cut-off, and the predicted value was defined based on whether 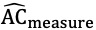 > cut-off. Accuracy, sensitivity, and specificity were computed for each participant and aggregated across participants (mean and SD).

##### Minute-Level Patterns of Daily Physical Activity

Minute-level activity count and 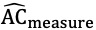 were used to estimate smoothed 24-hour time series of median activity count for age groups <60 years, 60-67 years, 68-74 years, and ≥75 years, for which 24-hour time series of median activity count have previously been published [25]. Activity count–based and 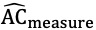-based estimates were compared by calculating MAPE defined as sum of absolute value of the difference between a pair of estimates divided by sum of activity count-based estimates.

## Results

### Population Characteristics

Data from 655 individuals (Table 1) were included in the analyses. The mean age was 69.8 (SD 14.2, range 22-97) years. There was a higher proportion of women (357/655, 54.5%) than men (298/655, 45.5%). The racial composition reflected that of the BLSA enrollment [2]. Of the 655 participants, 445 participants (67.9%) were White, 157 (24%) were Black, 44 (6.7%) were classified as other race, and 9 participants (1.4%) did not provide this information. Almost 96% of participants (628/655, 95.9%) self-reported good, very good, or excellent health. The prevalences of hypertension, high blood cholesterol levels, and osteoarthritis were 43.5% (285/655), 52.8% (346/655), and 48.2% (316/655), respectively. Participants had a median of 6 (range 3-7) days of valid accelerometry data; for valid days, participants had a mean of 1438 (SD 8) valid minutes (out of 1440 possible minutes per day).

The mean participant daily sums (Table 2) were 2,204,169 (SD 600,965) for activity count, 11,299.7 (SD 2766.0) for monitor-independent movement summary, 47.7 (SD 13.3) for mean amplitude deviation, 30.9 (SD 9.1) for Euclidean norm minus one, and 4157.6 (SD 1068.8) for activity intensity.

**Table 1:** Study sample (N=655) characteristics.

Characteristic					Value
**Sociodemographic**					
	**Age**				
		Mean (SD)	69.8 (14.2)		
		Median (range)	72.0 (22.0-97.0)		
	**Weight (kg)**				
		Mean (SD)	77.4 (17.1)		
		Median (range)	76.3 (41.1-142.7)		
	**Height (cm)**				
		Mean (SD)	168.0 (9.2)		
		Median (range)	167.3 (143.8-196.2)		
	**BMI**				
		Mean (SD)	27.3 (5.0)		
		Median (range)	26.6 (17.1-52.5)		
	**Sex**				
		Female count (%)	357 (54.5)		
		Male count (%)	298 (45.5)		
	**Race**				
		White count (%)	445 (67.9)		
		Black count (%)	157 (24.0)		
		Chinese count (%)	30 (4.6)		
		Hawaiian count (%)	11 (1.7)		
		Other non-White count (%)	3 (0.5)		
		Not reported count (%)	9 (1.4)		
**Sensor wear**					
	**Valid days**				
		Mean (SD)	5.9 (0.4)		
		Median (range)	6.0 (3.0, 7.0)		
	**Nonwear minutes (/day)**				
		Mean (SD)	2.0 (7.8)		
		Median (range)	0.0 (0.0, 77.0)		
	**Valid minutes (/day)**				
		Mean (SD)	1437.8 (8.0)		
		Median (range)	1440.0 (1361.7-1440.0)		
**Health**					
	**Self-reported health**				
		Good, very good, or excellent count (%)	628 (95.9)		
		Fair or poor count (%)	22 (3.4)		
		Not reported count (%)	5 (0.8)		
	**Medical history**				
		Myocardial infarction, congestive heart failure, ischemic chest pain, vascular procedure, or peripheral artery disease count (%)	55 (8.4)		
		Hypertension count (%)	285 (43.5)		
		High blood cholesterol count (%)	346 (52.8)		
		Stroke or transient ischemic attack count (%)	34 (5.2)		
		Pulmonary disease count (%)	74 (11.3)		
		Diabetes count (%)	95 (14.5)		
		Cancer count (%)	191 (29.2)		
		Osteoarthritis count (%)	316 (48.2)		

**Table 2:** Mean daily sum values for physical activity measures.

Measure			Value
**Activity count**			
	Mean (SD)	2,204,169 (600,965)	
	Median (range)	2,157,496 (731,945-5,071,196)	
**Monitor-independent movement summary**			
	Mean (SD)	11,299.7 (2766.0)	
	Median (range)	11,195.2 (4252.3-23,931.5)	
**Mean amplitude deviation**			
	Mean (SD)	47.7 (13.3)	
	Median (range)	46.3 (16.1-108.1)	
**Euclidean norm minus one**			
	Mean (SD)	30.9 (9.1)	
	Median (range)	29.6 (11.8-75.3)	
**Activity intensity**			
	Mean (SD)	4157.6 (1068.8)	
	Median (range)	4085.5 (1529.7-9418.6)	

### Correlations Between Minute-Level Summary Statistics

Monitor-independent movement summary was most correlated with activity count (estimated mean 0.988, SE 0.0002), closely followed by activity intensity (estimated mean 0.970, SE 0.0007, mean amplitude deviation (estimated mean 0.913, SE 0.0013), and Euclidean norm minus one (estimated mean 0.867, SE 0.0018) (Table 3).

The estimated effects of age (with female as the reference level) were not statistically significant in the models for activity count and monitor-independent movement summary (*P*=.97), activity count and mean amplitude deviation (*P*=.64), and activity count and activity intensity (*P*=.64), and were statistically significant in the model for activity count and Euclidean norm minus one (*P*<.001). The estimated effects of BMI on correlations were statistically significant for correlations between activity count and mean amplitude deviation (estimate 0.001, SE 0.0003, *P*=.001) and those between activity count and activity intensity (estimate 0.000278, SE 0.0001, *P*=.04). The estimated effects of sex (with female as the reference level) were statistically significant in the models for activity count and monitor-independent movement summary (estimate –0.002, SE 0.0005, *P*<.001), activity count and mean amplitude deviation (estimate –0.01, SE 0.0026, *P*<.001), and activity count and activity intensity (estimate –0.01, SE 0.0013, *P*<.001).

The results of secondary analysis (Table S1 in Multimedia Appendix 3) closely follow the results obtained from the full sample (Table 3) for both unadjusted (activity count and monitor-independent movement summary: difference 0; activity count and Euclidean norm minus one: difference –0.06; activity count and mean amplitude deviation: difference 0.02; activity count and activity intensity: difference 0.01) and adjusted models.

**Table 3:** Summary of intercept-only linear regression and adjusted linear regression with outcome defined as participant-specific correlation between activity count and other measures (monitor-independent movement summary, Euclidean norm minus one, mean amplitude deviation, or activity intensity).

Model and response variable^a^			Intercept		Age				BMI				Sex^b^		
		Estimate (SE)		Estimate (SE)		*P* value		Estimate (SE)		*P* value		Estimate (SE)		*P* value	
**Unadjusted**															
	Monitor-independent movement summary	0.988042 (0.000232)		—^c^		—		—		—		—		—	
	Euclidean norm minus one	0.867158 (0.001841)		—		—		—		—		—		—	
	Mean amplitude deviation	0.913412 (0.001320)		—		—		—		—		—		—	
	Activity intensity	0.969984 (0.000687)		—		—		—		—		—		—	
**Adjusted for age, BMI, and sex**															
	Monitor-independent movement summary	0.987969 (0.001744)		0.000001 (0.000016)		.97		0.000032 (0.000046)		.48		–0.001859 (0.000466)		<.001	
	Euclidean norm minus one	0.886566 (0.013766)		–0.000532 (0.000129)		<.001		0.000653 (0.000363)		.07		–0.000206 (0.003678)		.96	
	Mean amplitude deviation	0.892177 (0.009852)		0.000044 (0.000092)		.64		0.000840 (0.000260)		.001		–0.010410 (0.002632)		<.001	
	Activity intensity	0.962364 (0.005016)		0.000063 (0.000047)		.18		0.000278 (0.000132)		.04		–0.009576 (0.001340)		<.001	

^a^Correlation with activity count.

^b^Female was used as the reference.

^c^Not included in the model.

### Mapping Between Minute-Level Summary Measures

#### Model Fit

**Figure 1:**
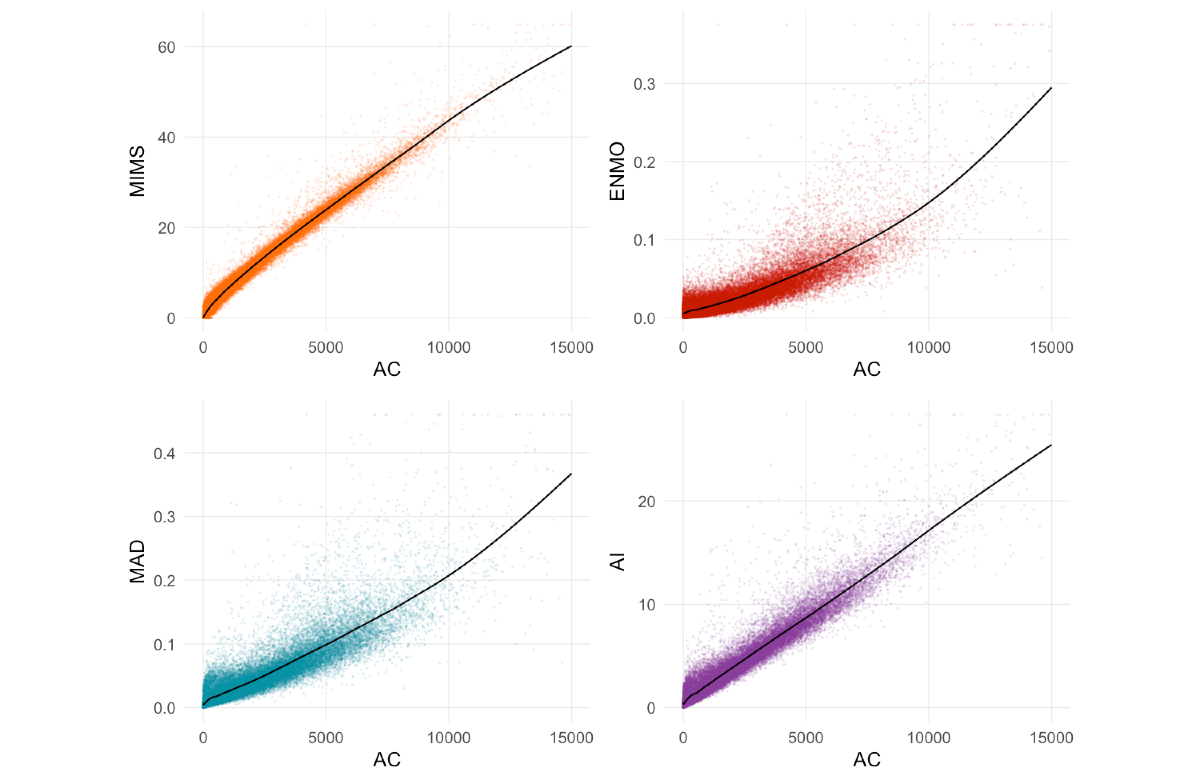
Estimated minute-level mapping. A black solid line shows generalized additive model–fitted values of a measure (monitor-independent movement summary, Euclidean norm minus one, mean amplitude deviation, activity intensity) given the activity count value. The points represent a subset of the data created by taking every 100th observations from all participant- and minute-specific observations; this subset is the same for all 4 plots. AC: activity count; AI: activity intensity; ENMO: Euclidean norm minus one; MAD: mean amplitude deviation; MIMS: monitor-independent movement summary.

Figure 1 shows the estimated association between minute-level activity count (x-axis) and minute-level monitor-independent movement summary, Euclidean norm minus one, mean amplitude deviation, and activity intensity (y-axis). The black solid line represents fitted values obtained from generalized additive models.

For a widely used activity count cut-off 1853 [23], the corresponding cut-offs (Table 4) were 10.558 (monitor-independent movement summary), 0.022 (Euclidean norm minus one), 0.039 (mean amplitude deviation), and 3.620 (activity intensity).

**Table 4:** Corresponding values of each measure for activity count cut-off values.

Method	Activity count cut-off value	Corresponding value			
		Monitor-independent movement summary	Euclidean norm minus one	Mean amplitude deviation	Activity intensity
Separate sedentary and active in older adults [23]	1853	10.558	0.022	0.039	3.620
Separate sedentary and light activity in young to older adults [24]	2860	15.047	0.033	0.057	5.273
Separate light and moderate-to-vigorous activity in young to older adults [24]	3940	19.614	0.046	0.078	7.025

#### Mapping Evaluation

In the task of estimating total activity count, MAPE values were lowest for monitor-independent movement summary (mean 2.5, SD 2.4), followed by activity intensity (mean 6.3, SD 5.1), mean amplitude deviation (mean 11.3, SD 8.4), and Euclidean norm minus one (mean 14.3, SD 10.3). MPE values were similar for monitor-independent movement summary (mean 0.2, SD 3.2), activity intensity (mean 0.3, SD 7.6), mean amplitude deviation (mean –0.3, SD 13.3), and Euclidean norm minus one (mean 4.6, SD 16.1). The findings for median absolute percentage error and median percentage error were similar to those for MAPE and MPE, respectively (Table S2 in Multimedia Appendix 3).

Based on visual inspection, there was larger variability in MPE values among participants with smaller mean total activity count values, but there was no apparent tendency for lower or higher MPE values based on participants’ average total activity counts (Figure S1 in Multimedia Appendix 3).

In the task of predicting whether the activity count for a given minute was above a certain cut-off, for the cut-off equal 1853, participant-specific classification accuracy (Table S3 in Multimedia Appendix 3) was the highest for monitor-independent movement summary (mean 0.981, SD 0.005), followed by activity intensity (mean 0.960, SD 0.012), mean amplitude deviation (mean 0.928, SD 0.021), and Euclidean norm minus one (mean 0.904, SD 0.028). Overall, the accuracy of predicting whether the activity count for a given minute was above a certain cut-off was better for higher activity count cut-off values (ie, accuracy was higher for predicting whether a given minute has activity count >3940 than for predicting whether a given minute activity count >2860; Table S3 in Multimedia Appendix 3). This is consistent with our observation that the variability along the estimated mapping is lower for higher activity values (Figure S1 in Multimedia Appendix 3).

#### Minute-Level Patterns of Daily Physical Activity

**Figure 2:**
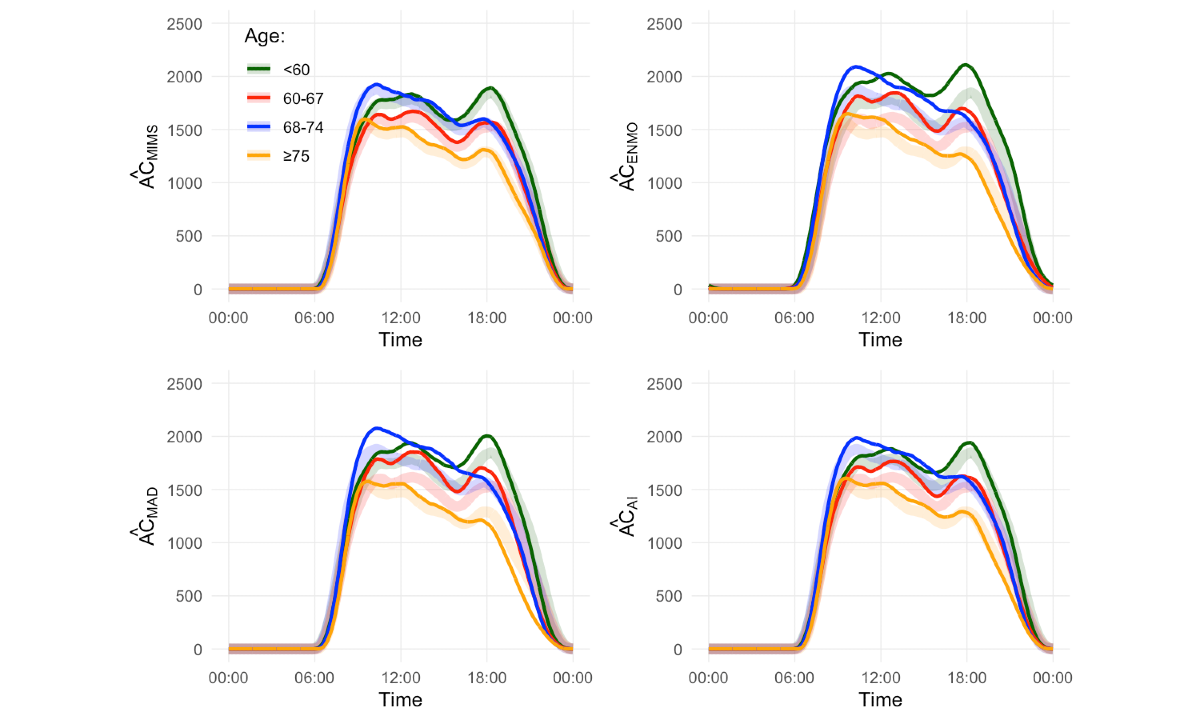
Smoothed 24-hour median activity counts per minute for each age group: <60 years (green), 60-67 years (red), 68-74 years (blue), and ≥75 years (orange). Semitransparent thick colored lines represent results obtained with activity count; they are the same for all 4 plots. Solid thin colored lines represent results obtained with values mapped into activity count from monitor-independent movement summary, Euclidean norm minus one, mean amplitude deviation, or activity intensity. AC: activity count; AI: activity intensity; ENMO: Euclidean norm minus one; MAD: mean amplitude deviation; MIMS: monitor-independent movement summary.

Figure 2 shows the estimated smoothed 24-hour median activity counts across the previously published age groups: <60-year old (green; N = 140), 60- to 67-year old (red; N = 102), 68- to 74-year old (blue; N = 129), ≥ 75-year old (orange; N = 284). Semi-transparent thick colour lines represent results obtained with activity count.Solid thin colour lines represent results obtained with 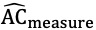.

The 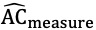-based curves yielded roughly the same information as the activity count-based curves [25] for each age group (<60 years: n=140; 60-67 years: n=102; 68-74 years: n=129; ≥75 years: n=284). MAPE for activity count-based and 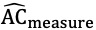-based estimates was the lowest for monitor-independent movement summary (MAPE 3.2), followed by activity intensity (MAPE 6.7), mean amplitude deviation (MAPE 11.1), and Euclidean norm minus one (MAPE 12.5).

## Discussion

### Principal Results

Correlations between activity count and the other raw data summary metrics were all large (mean *r*≥0.87) and were especially high for monitor-independent movement summary and activity intensity (mean *r*≥0.97) (Table 3). After harmonization, monitor-independent movement summary allowed for excellent accuracy in predicting total activity count and sedentary minutes using a cut-off that corresponded to an activity count cut-off determined using [23]. Our analysis is especially timely given the recent release of physical activity data from NHANES 2011-2014 that uses the open-source monitor-independent movement summary measure.

To the best of our knowledge, the correlation between activity count and monitor-independent movement summary in continuous data collected in the free-living environment has not been previously explored. The activity count measure had the highest mean participant-specific correlation with monitor-independent movement summary (mean *r*= 0.988), closely followed by activity intensity (mean *r*=0.97), and mean amplitude deviation (mean *r*=0.913) and Euclidean norm minus one (mean *r*=0.867). Both monitor-independent movement summary and activity intensity measures are based on variability within each dimension, whereas mean amplitude deviation and Euclidean norm minus one are based on the Euclidean norm of three-dimensional data; therefore, it is consistent with expectations that monitor-independent movement summary and activity intensity behave similarly and demonstrate similar correlations with activity count. While we found there were statistically significant effects of age (in the model for correlation between activity count and Euclidean norm minus one: *P*<.001), BMI (in the model for correlation between activity count and mean amplitude deviation: *P*=.001; in the model for correlation between activity count and activity intensity: *P*=.04), and sex (in the model for correlation between activity count and monitor-independent movement summary: *P*<.001; in the model for activity count and mean amplitude deviation: *P*<.001; in the model for activity count and activity intensity: *P*<.001), the effect sizes were of very small magnitude. In particular, the analysis showed that monitor-independent movement summary had a correlation with activity count that did not differ significantly for age (*P*=.97) or BMI (*P*=.48), and differed significantly (*P*<.001) between men and women by a magnitude of 0.002. The results from secondary analysis, with a subsample of the youngest participants (participants of age 65 years or less; 31.9% of the full sample), were similar to those from the full sample.

Harmonization mapping can be particularly useful to translate commonly used cut-off values of physical activity intensity levels from activity count into measures implemented in open-source software. For the tasks of predicting sedentary minutes for an activity count cut-off of 1853 [23], we observed excellent accuracy for monitor-independent movement summary (accuracy 0.981) and activity intensity (accuracy 0.960). The utility of the derived mapping was demonstrated in the example in which previous findings [25] were replicated. The physical activity volume daily trajectories for age groups obtained with activity count were closely matched with those from the measures, with monitor-independent movement summary yielding visually almost identical results (MAPE 3.2), followed by activity intensity (MAPE 6.7), mean amplitude deviation (MAPE 11.1), and Euclidean norm minus one (MAPE 12.5).

To the best of our knowledge, we are the first to provide freely available R software (SummarizedActigraphy R package) with a unified interface for computation of the 4 open-source measures from raw accelerometry data, with complicated mathematical formulas distilled into a reader-friendly text (Multimedia Appendix 2).

### Limitations

First, the data were from a sample that consisted of predominantly middle-aged to older adults (Table 1). However, we observed that (1) the level of activity of adults in the sample ranged from sedentary to moderate and vigorous activity, (2) mapping results did not exhibit any trend based on the average level of the participant's physical activity, and (3) the variability of estimates was lower for higher activity values, which suggests that mapping could prove useful in future studies with younger (more active) populations [25].

Second, physical activity measures were computed using raw accelerometry data collected at a frequency of 80 Hz. While this frequency matches that of physical activity data from NHANES 2011-2014 [12] that uses the monitor-independent movement summary measure, caution should be used in adapting our harmonization mapping to raw data collected at a different frequency.

Third, data had been collected with sensors worn on the nondominant wrist only. While we expect the results to be generalizable to data from sensors worn on the dominant wrist, we presume that correlations and mapping would not be applicable to chest- or hip-worn sensors, because physical activity volume statistics (eg, total activity count) calculated from raw data collected by these devices are expected to be substantially lower than when measured at wrist.

Fourth, harmonization mapping was estimated using generalized additive modeling, which does not offer an easy, closed-form formula of the transformation. While a closed-form formula could be obtained using polynomial regression models, the choice of generalized additive models allowed for thorough estimation of a relationship between activity count and other measures in a more flexible way.

Finally, our results may be conditional upon the data preprocessing methods used; however, we believe that the steps we performed are commonly done [17,19] and are reasonable given the obtained data summary statistics and visual quality checks performed.

### Conclusions

Activity count was highly correlated with monitor-independent movement summary, Euclidean norm minus one, mean amplitude deviation, and activity intensity. Mapping provides a way to harmonize accelerometry data sets with different summary measures; however, further research is warranted to test the validity of mapping with data collected at a different frequency or from different body locations.

## Appendix

Multimedia Appendix 1 Raw accelerometry data quality control.

Multimedia Appendix 2 Open-source summary measures of raw accelerometry data.

Multimedia Appendix 3 Results.

## Abbreviations

BLSA: Baltimore Longitudinal Study on Aging
MAPE: mean absolute percentage error
MPE: mean percentage error
NHANES: National Health and Nutrition Examination Survey

## Data Availability

Data used in this manuscript were collected as part of the National Institute on Aging’s Baltimore Longitudinal Study of Aging. A project repository with all data preprocessing and analysis code is publicly available on GitHub (muschellij2/blsa_mims).
