# Supplementary material for: Comparison of Accelerometry-Based Measures of Physical Activity: Retrospective Observational Data Analysis Study
Source: JMIR Mhealth Uhealth. 2022 Jul 22;10(7):e38077. doi: 10.2196/38077 (PMC9356340; doi:10.2196/38077)
Supplement: Multimedia Appendix 1 [file mhealth_v10i7e38077_app1.docx]

# Appendix 1. Raw Accelerometry Data Quality Control

Three raw data quality check flags were adapted from a set of nine flags recently introduced by the release of the NHANES 2011-2012 wave protocol [1]. The selected flags subset represents intuitive flags that are meant to "determine signal patterns that were unlikely to be a result of human movement" but are not aimed at identifying non-wear. To provide the flags definition, we denote a raw data observation as a vector $\mathbf{x}(t)=(x_{1}(t),x_{2}(t),x_{3}(t))$, where $x_{m}(t)$ is an acceleration measurement along axis $m=1,2,3$ at time $t$.

First, large changes in acceleration values ("spikes") were identified. Specifically, an observation $\mathbf{x}(t)$ was flagged if for any axis $m=1,2,3$, $x_{m}(t)$ had an adjacent observation, $x_{m}(t-1)$ or $x_{m}(t+1)$, with an absolute difference greater than a threshold of 14.7 *g*. The 14.7 *g* threshold was adapted from NHANES protocol (11 *g*), as the NHANES devices had a dynamic range of 12 *g*, and the BLSA devices had a range of 16 *g*, so our threshold is proportional (11/12) to our data. Second, an observation $\mathbf{x}(t)$ was flagged if any axis measurement $x_{m}(t)$ occurred near the device maximum limit (here: 8 *g*, NHANES protocol: 6 *g*) with a tolerance margin (0.05 *g*), which translates to $x_{m}(t)$ being equal to or greater than 7.95 *g*. Third, an observation $\mathbf{x}(t)$ was flagged if any axis measurement $x_{m}(t)$ occurred near the device minimum limit (here: -8 *g*, NHANES protocol: -6 *g*), including a tolerance margin (0.05 *g*), and had same-axis adjacent observation also near the device minimum limit. These three flags were combined and the raw data observation $\mathbf{x}(t)$ was flagged as valid if it had none of the three flags; otherwise it was invalid.

## References

1. NHANES 2011-2012 Data Documentation (2020). National Health and Nutrition Examination Survey 2011-2012. Data Documentation, Codebook, and Frequencies: Quality Assurance and Quality Control. Available from: https://wwwn.cdc.gov/Nchs/Nhanes/2011-2012/PAXMIN_G.htm. Last accessed on 2022-07-05.
