# Supplementary material for: Comparison of Accelerometry-Based Measures of Physical Activity: Retrospective Observational Data Analysis Study
Source: JMIR Mhealth Uhealth. 2022 Jul 22;10(7):e38077. doi: 10.2196/38077 (PMC9356340; doi:10.2196/38077)
Supplement: Multimedia Appendix 2 [file mhealth_v10i7e38077_app2.docx]

# Appendix 2. Open-source Summary Measures of Raw Accelerometry Data

The raw accelerometry data were used to derive a set of commonly used minute-level open-source summary measures: MIMS, ENMO, MAD, and AI. To provide the measures definition, we denote a raw data observation as a vector $\mathbf{x}(t)=(x_{1}(t),x_{2}(t),x_{3}(t))$, where $x_{m}(t)$ is an acceleration measurement along $m=1,2,3$ axis collected at time $t$.

## Monitor Independent Movement Summary (MIMS)

[1] proposed Monitor-Independent Movement Summary unit (MIMS-unit). The MIMS-unit algorithm steps are conducted independently for each axis' univariate acceleration signal $x_{m}(t), m=1,2,3$ until a final aggregation step. First, an input signal $x_{m}(t)$ is extrapolated to address a possible case when detected acceleration exceeds a sensor’s dynamic range; in this procedure, $x_{m}(t)$ is interpolated to 100 Hz, and then the extrapolation algorithm is applied to identify observations that hit the device limit (here: ± 8 *g*) and replace them with spline-interpolated points derived from the estimated extrapolation peak. The rest of the computations are done on this 100 Hz data. Second, a fourth-order Butterworth bandpass filter (0.2-5 Hz) is applied. Third, the interpolated, extrapolated, and filtered signal, $x_{m}^{(f)}(t)$, is aggregated within an epoch by computing area under curve via numerical integration; here, the epoch was set to 1 minute. Fourth, integrated values from each of the three axes are summed, yielding one value per epoch. Finally, the values less than or equal to 0.0001 * (epoch in seconds) * (sample rate after interpolation) (here: 0.0001 * 60 * 100 = 0.6) are truncated to zero.

The MIMS procedure may produce a negative value (-0.01), which indicates "the algorithm is unable to output a valid MIMS value for the given piece of the signal" (see Issues section on GitHub repository mHealthGroup/MIMSunit). Negative MIMS output values were set to missing observations.

The algorithm implementation is provided in the MIMSunit R package. To compute MIMS, the package's method mims_unit was used with its default values of internal parameters (consistent with the manuscript recommendations).

## Euclidean Norm Minus One (ENMO)

[2] proposed Euclidean Norm Minus One (ENMO) summary measure. ENMO calculation is based on Euclidean norm of $(x_{1}(t),x_{2}(t),x_{3}(t))$,

$$r(t)=\sqrt{x_{1}^{2}(t)+x_{2}^{2}(t)+x_{3}^{2}(t)},$$

where negative values after subtracting are set to zero. Explicitly, the ENMO measure per epoch of $H$ observations starting at time $t_{0}$ is defined as

$ENMO(t_{0};H)=\frac{1}{H}\Sigma_{h=0}^{H-1}max\{r(t_{0}+h)-1,0\}$.

Here, $H$ of size 60 * 80 = 4800 observations was used to match the number of observations in one minute with frequency of our raw accelerometry data. For each minute, $t_{0}$ was set to be the time of the first observation within that minute.

Following the recommendations from [5], raw data calibration was performed for each participant separately before computing the ENMO measure. The g.calibrate method from the GGIR R package was used to estimate calibration values that were further used to center and scale the data accordingly. No other measures used the post-calibrated data.

## Mean Amplitude Deviation (MAD)

[3] introduced Mean Amplitude Deviation (MAD) as a summary measure for accelerometry data. MAD measure per epoch of $H$ observations starting at time $t_{0}$ is defined as

$MAD(t_{0};H)=\frac{1}{H}\Sigma_{h=0}^{H-1}|r(t_{0}+h)-\bar{r}(t_{0};H)|$,

where $\bar{r}(t_{0};H)$ is defined as average Euclidean norm in the epoch, formally

$\bar{r}(t_{0};H)=\frac{1}{H}\Sigma_{h=0}^{H-1}r(t_{0}+h)$.

Here, $H$ and $t_{0}$ values were defined the same as for ENMO.

## Activity Index (AI)

[4] proposed the (unnormalized) Activity Index (AI) measure based on the combination of the three within-axis variance statistics. The variance of acceleration along $m$-th axis in the window of length $H$starting at time $t_{0}$ is defined as

$$\sigma_{m}^{2}(t_{0};H)=\frac{1}{H}\sum_{h=0}^{H-1} [x_{m}(t_{0}+h)-\mu_{m}(t_{0};H)]^{2}, m=1,2,3,$$

where $\mu_{m}(t_{0};H)$ is axis-specific mean acceleration in the window, formally

$$\mu_{m}(t_{0};H)=\frac{1}{H}\sum_{h=0}^{H-1} x_{m}(t_{0}+h), m=1,2,3.$$

Then AI measure per epoch of $H$ observations starting at time $t_{0}$ is originally defined as

$AI(t_{0};H)=\sqrt{max\{\frac{1}{3}[\Sigma_{m=1}^{3}\sigma_{m}^{2}(t_{0};H) -\bar{\sigma}^{2}],0\}}$,

where $\bar{\sigma}^{2}$ is the systematic noise variance calculated using the data collected during some non-moving period. In our work, $\bar{\sigma}^{2}$ is not estimated and is set to zero in the above equation; hence, the AI formula used narrows down to

$AI(t_{0};H)=\sqrt{max\{\frac{1}{3}\Sigma_{m=1}^{3}\sigma_{m}^{2}(t_{0};H),0\}}$.

In computation of AI, first, a window $H$ of size 1 * 80 = 80 was used to match the number of observations in one second with frequency of our raw accelerometry data, and $t_{0}$ was set to be the time of the first observation within each second. Next, the per-second AI values were summed up within each minute so as the final outcome is defined at the minute level. This procedure is consistent with the recommendations given in [6].

## References

1. John, D., Tang, Q., Albinali, F., Intille, S. (2019). An Open-Source Monitor-Independent Movement Summary for Accelerometer Data Processing. Journal for the Measurement of Physical Behaviour, 2(4), 268–281. doi:10.1123/jmpb.2018-0068
2. Van Hees, V. T., Gorzelniak, L., Leon, E. C. D., Eder, M., Pias, M., Taherian, S., Ekelund, U., Renström, F., Franks, P. W., Horsch, A., Brage, S. (2013). Separating movement and gravity components in an acceleration signal and implications for the assessment of human daily physical activity. PLoS ONE, 8(4):e61691. doi:10.1371/journal.pone.0061691
3. Vähä-Ypyä, H., Vasankari, T., Husu, P., Suni, J., Sievänen, H. (2015). A universal, accurate intensity-based classification of different physical activities using raw data of accelerometer. Clinical Physiology and Functional Imaging, 35(1), 64–70. doi:10.1111/cpf.12127
4. Bai, J., He, B., Shou, H., Zipunnikov, V., Glass, T. A., Crainiceanu, C. M. (2014). Normalization and extraction of interpretable metrics from raw accelerometry data. Biostatistics (Oxford, England), 15(1), 102–116. doi:10.1093/biostatistics/kxt029
5. Van Hees, V. T., Fang, Z., Langford, J., Assah, F., Mohammad, A., da Silva, I. C. M., Trenell, M. I., White, T., Wareham, N. J., Brage, S. (2014). Autocalibration of accelerometer data for free-living physical activity assessment using local gravity and temperature: an evaluation on four continents. Journal of Applied Physiology (1985), 117(7), 738–744. doi:10.1152/japplphysiol.00421.2014
6. Bai, J., Di, C., Xiao, L., Evenson, K. R., LaCroix, A. Z., Crainiceanu, C. M., Buchner, D. M. (2016). An Activity Index for Raw Accelerometry Data and Its Comparison with Other Activity Metrics. PloS One, 11(8), e0160644–e0160644. doi:10.1371/journal.pone.0160644
