# Supplementary material for: Comparison of Accelerometry-Based Measures of Physical Activity: Retrospective Observational Data Analysis Study
Source: JMIR Mhealth Uhealth. 2022 Jul 22;10(7):e38077. doi: 10.2196/38077 (PMC9356340; doi:10.2196/38077)
Supplement: Multimedia Appendix 3 [file mhealth_v10i7e38077_app3.docx]

# Appendix 3. Results

Table S1. Summary of Pearson correlation for pairs of minute-level measures: AC, MIMS, ENMO, MAD, AI, computed using a subsample of participants of age 65 years or less (N = 209; 31.9% of the full sample). In all models, participant-specific value of correlation was set as an outcome. The "*" symbol is used to denote model coefficients (excluding intercept) for which the corresponding *P* was less than .05.

|  | Unadjusted model | Model adjusted for: age, BMI, sex | | | |
| --- | --- | --- | --- | --- | --- |
|  | Intercept | Intercept | Age | BMI | Sex^a^ |
|  | Est. (SE)^c^ | Est. (SE) | Est. (SE) | Est. (SE) | Est. (SE) |
|  |  |  |  |  |  |
| **Response var.** ^b^ |  |  |  |  |  |
| cor(AC,MIMS) | 0.988 (0.0005) | 0.989 (0.0031) | < 0.001 (<0.0001) | < 0.001 (0.0001) | -0.002 (0.0009)* |
| cor(AC,ENMO) | 0.873 (0.0028) | 0.862 (0.0192) | > -0.001 (0.0003) | 0.001 (0.0005) | 0.007 (0.0058) |
| cor(AC,MAD) | 0.911 (0.0024) | 0.903 (0.0162) | > -0.001 (0.0002) | 0.001 (0.0004)* | -0.011 (0.0049)* |
| cor(AC,AI) | 0.969 (0.0015) | 0.965 (0.0098) | > -0.001 (0.0001) | < 0.001 (0.0003) | -0.012 (0.0030)* |

^a^Sex = Sex covariate with female set as reference.

^b^Response var. = Response variable in a model.

^c^Est. (SE) = Model coefficient estimate (model coefficient standard error).

Table S2. Summary of participant-specific error in estimating total activity counts (TAC): mean percentage error (MPE), mean absolute percentage error (MAPE), median percentage error (MdPE), median absolute percentage error (MdAPE).

|  | MPE | MAPE | MdPE | MdAPE |
| --- | --- | --- | --- | --- |
|  | Mean (SD) | Mean (SD) | Mean (SD) | Mean (SD) |
|  |  |  |  |  |
| **TAC estimate based on** |  |  |  |  |
| $\hat{AC}_{\mathrm{MIMS}}$ | 0.2 (3.2) | 2.5 (2.4) | 0.1 (3.2) | 2.3 (2.3) |
| $\hat{AC}_{\mathrm{ENMO}}$ | 4.6 (16.1) | 14.3 (10.3) | 3.9 (16.2) | 13.4 (10.5) |
| $\hat{AC}_{\mathrm{MAD}}$ | -0.3 (13.3) | 11.3 (8.4) | -0.8 (13.4) | 10.7 (8.7) |
| $\hat{AC}_{\mathrm{AI}}$ | 0.3 (7.6) | 6.3 (5.1) | -0.1 (7.6) | 5.8 (5.3) |


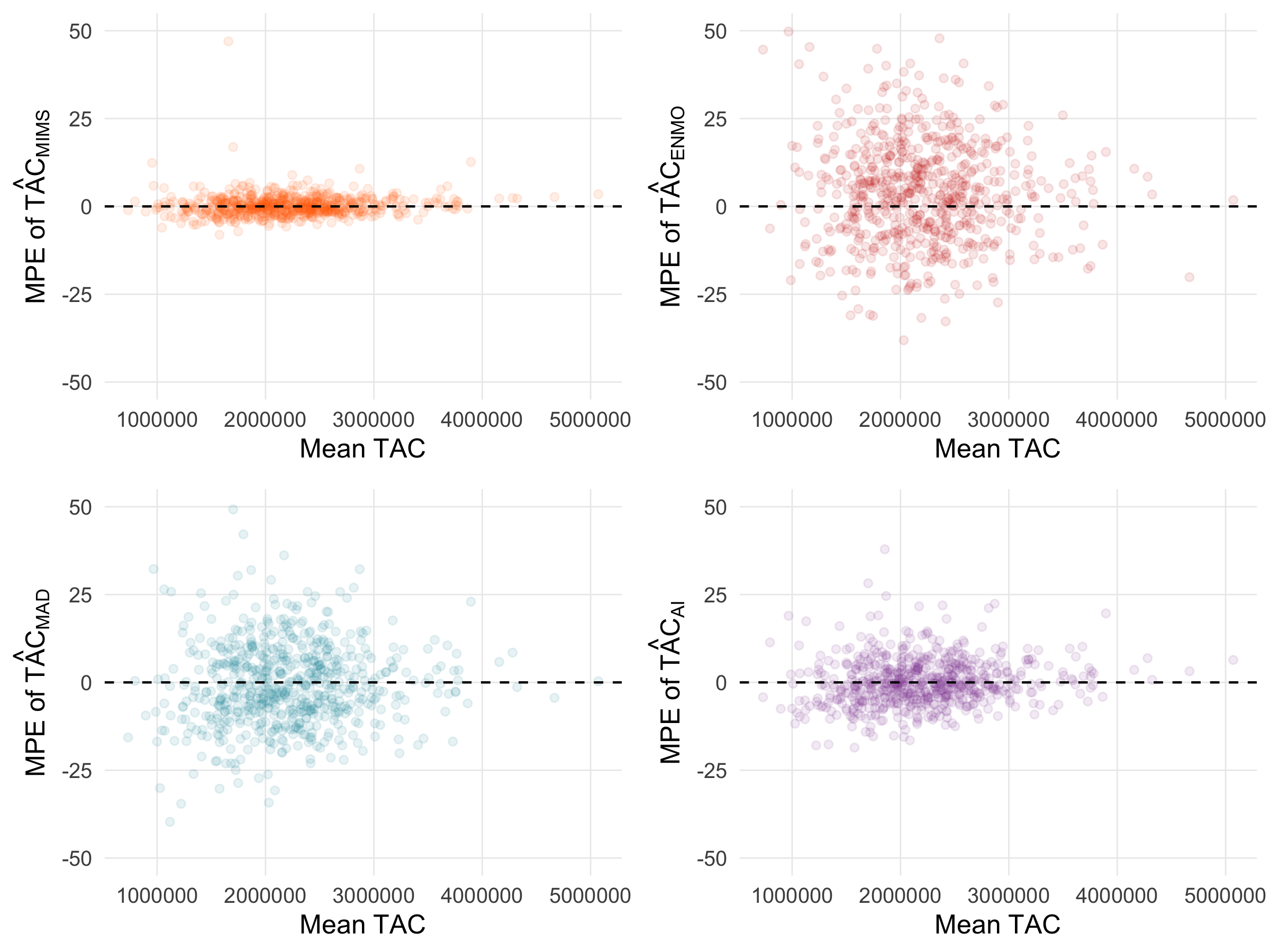


Figure S1. Participant-specific mean percentage error (MPE) in estimating total activity counts (TAC), arranged according to the participant's average TAC. Each point represents one participant's MPE.

Table S3. Summary of participant-specific accuracy, sensitivity and specificity of predicting whether a minute is above an AC cut-off: AC = 1853, AC = 2860 and 3940*.* The minute's label was based on true AC and the prediction was based on ${\hat{\mathrm{AC}}}_{\mathrm{measure}}$. For each participant, performance metrics -- accuracy, sensitivity and specificity -- were computed across all participant's minutes. Columns 3-5 show mean and standard deviation of participant-specific performance metrics.

|  |  | Accuracy | Sensitivity | Specificity |
| --- | --- | --- | --- | --- |
|  |  | Mean (SD) | Mean (SD) | Mean (SD) |
|  |  |  |  |  |
| **Min.'s label**^a^ | **Min.'s pred.** ^b^ |  |  |  |
| AC > 1853 | ${\hat{\mathrm{AC}}}_{\mathrm{MIMS}}$> 1853 | 0.981 (0.005) | 0.968 (0.012) | 0.986 (0.007) |
| AC > 1853 | ${\hat{\mathrm{AC}}}_{\mathrm{ENMO}}$> 1853 | 0.904 (0.028) | 0.856 (0.071) | 0.921 (0.043) |
| AC > 1853 | ${\hat{\mathrm{AC}}}_{\mathrm{MAD}}$> 1853 | 0.928 (0.021) | 0.879 (0.061) | 0.945 (0.028) |
| AC > 1853 | ${\hat{\mathrm{AC}}}_{\mathrm{AI}}$> 1853 | 0.960 (0.012) | 0.933 (0.030) | 0.970 (0.018) |
| AC > 2860 | ${\hat{\mathrm{AC}}}_{\mathrm{MIMS}}$> 2860 | 0.985 (0.004) | 0.963 (0.017) | 0.990 (0.005) |
| AC > 2860 | ${\hat{\mathrm{AC}}}_{\mathrm{ENMO}}$> 2860 | 0.930 (0.022) | 0.820 (0.106) | 0.957 (0.027) |
| AC > 2860 | ${\hat{\mathrm{AC}}}_{\mathrm{MAD}}$> 2860 | 0.941 (0.018) | 0.836 (0.095) | 0.966 (0.019) |
| AC > 2860 | ${\hat{\mathrm{AC}}}_{\mathrm{AI}}$ > 2860 | 0.967 (0.010) | 0.914 (0.048) | 0.980 (0.013) |
| AC > 3940 | ${\hat{\mathrm{AC}}}_{\mathrm{MIMS}}$ > 3940 | 0.986 (0.004) | 0.945 (0.032) | 0.992 (0.005) |
| AC > 3940 | ${\hat{\mathrm{AC}}}_{\mathrm{ENMO}}$ > 3940 | 0.941 (0.020) | 0.743 (0.148) | 0.970 (0.020) |
| AC > 3940 | ${\hat{\mathrm{AC}}}_{\mathrm{MAD}}$ > 3940 | 0.946 (0.017) | 0.753 (0.137) | 0.974 (0.015) |
| AC > 3940 | ${\hat{\mathrm{AC}}}_{\mathrm{AI}}$ > 3940 | 0.969 (0.010) | 0.870 (0.079) | 0.983 (0.011) |

^a^Min.s' label = condition defining a minute's true label in the classification task.

^b^Min.s' pred. = condition defining a minute's predicted label in the classification task.
